# New Books

**Published:** 1872-06

**Authors:** 


NEW BOOKS.—Authors and Publishers have been busily engaged during the past winter in getting their works ready for the public, and the result has been some very valuable and interesting works which have already appeared and others which will be ready in a short time.

Messrs Lindsay & Blakiston are about to publish several valuable and instructive works among which we notice a new edition of "Hewitts Diagnosis and Treatment of diseases preculiar to Women, and Clarks outlines of Surgery."

Wm. Wood & Co. announce a new edition of Holmes' System ot Surgery in five volumes, illustrated. They will also shortly publish among others a new edition of Byford's practice of Obstetrics and of Loomis' Physical Diagnosis.

Among the works now going through the press of Messrs Lippincott & Co. we notice a work on Ovarian Tumors by W. L. Atlee, M. D. and Beck's Medical Jurisprudence.

A new edition of Watson's lectures on the Principles and Practice of Physic, and of Gross' System of Surgery, is to be published shortly by Henry C. Lea of Philadelphia. Mr. Lea has also in preparation several other valuable works on medicine and surgery.

Messrs D. Appleton & Co. have several medical works in press, including a treatise on Ovarian Tumors. By E. R. Peaslee, M. D., LL. D. A work on Diseases of the Ovaries. By Dr. T. Spencer Wells. And a Treatise on the Surgical diseases of the male genito-urinary organs, including syphilis. By W. H. Van Buren, A. M., M, D. and Edward L. Keyes, A. M., M. D.

